# The digestive tract as the origin of systemic inflammation

**DOI:** 10.1186/s13054-016-1458-3

**Published:** 2016-10-18

**Authors:** Petrus R. de Jong, José M. González-Navajas, Nicolaas J. G. Jansen

**Affiliations:** 1 Department of Pediatric Intensive Care, Wilhelmina Children’s Hospital, University Medical Center Utrecht, Utrecht, The Netherlands; 2 Sanford Burnham Prebys Medical Discovery Institute, 10901 N Torrey Pines Rd, La Jolla, CA 92037 USA; 3 Networked Biomedical Research Center for Hepatic and Digestive Diseases (CIBERehd), Hospital General Universitario de Alicante, Alicante, Spain; 4 Alicante Institute of Health and Biomedical Research (ISABIAL – FISABIO Foundation), Alicante, Spain

**Keywords:** Acute inflammation, Gastrointestinal failure, Gut-liver crosstalk, Pancreatitis

## Abstract

Failure of gut homeostasis is an important factor in the pathogenesis and progression of systemic inflammation, which can culminate in multiple organ failure and fatality. Pathogenic events in critically ill patients include mesenteric hypoperfusion, dysregulation of gut motility, and failure of the gut barrier with resultant translocation of luminal substrates. This is followed by the exacerbation of local and systemic immune responses. All these events can contribute to pathogenic crosstalk between the gut, circulating cells, and other organs like the liver, pancreas, and lungs. Here we review recent insights into the identity of the cellular and biochemical players from the gut that have key roles in the pathogenic turn of events in these organ systems that derange the systemic inflammatory homeostasis. In particular, we discuss the dangers from within the gastrointestinal tract, including metabolic products from the liver (bile acids), digestive enzymes produced by the pancreas, and inflammatory components of the mesenteric lymph.

## Background

Gastro-intestinal (G-I) pathology in the critically ill patient mainly involves the small intestine. The inner lining of the small intestine is covered by a single layer of intestinal epithelial cells (IECs) and is organized as crypts and villi. The crypts are mostly made up of proliferating cells, whereas the villi are covered by fully matured epithelial cells to provide 200–300 m^2^ of absorptive surface [1]. All IEC types derive from intestinal stem cells that reside at the crypt bottom [2]. Precursor cells differentiate into absorptive enterocytes or one of the secretory cell types. Enterocytes are equipped with microvilli and express nutrient transporters to maximize the uptake of solutes, simple carbohydrates, and amino acids. Secretory cells include mucus-producing Goblet cells, antimicrobial peptide-producing Paneth cells, and hormone-producing enteroendocrine cells [3]. Products released by secretory cell types are vital for the maintenance of the gut barrier and motility. Paneth cells provide a protective niche for intestinal stem cells and maintain homeostatic host–microbial interactions [4]. These cellular systems provide fail-safe mechanisms to ensure continuous turnover of epithelial cells and maintenance of the intestinal barrier.

The commensal microflora in a healthy gut constitute approximately 10^3^–10^4^ different bacterial species, mostly of the *Firmicutes* and *Bacteroidetes* phyla. They are indispensable for the digestion of dietary substrates, exert pro-proliferative effects on IECs, promote enterocyte differentiation, prevent colonization of pathogens, and educate the mucosal and systemic immune system [5]. Furthermore, our microbiota is involved in the generation of secondary bile acids, which promote the uptake of dietary lipids and fat-soluble vitamins [6]. The fermentation of complex carbohydrates yields short-chain fatty acids (SCFAs; e.g., butyrate) that serve as an energy source for the host and display beneficial effects on immune cells [5], IEC proliferation, differentiation, and gut barrier function [7]. Importantly, SCFAs also mediate anti-inflammatory effects on immune cells, which involves signaling via G-protein-coupled receptor 41 (GPR41) and GPR43. GPR43 signaling is anti-inflammatory in the gut [8]. However, GPR43 deficiency results in increased mortality upon gut barrier loss, most likely due to septic complications of bacterial translocation associated with aberrant neutrophil chemotaxis [9]. Thus, the intestinal microbiota and its metabolic products are vital for gut homeostasis.

Systemic stress, such as major trauma, burns, or surgery, can disturb this delicate balance, leading to epithelial denudation of villi, enterocyte dysfunction, gut barrier loss, and translocation of luminal constituents to the circulation [10]. This may occur with only mild systemic inflammation, for example, leakage of endotoxins (lipopolysaccharide (LPS)) from the intestinal lumen to the circulation occurs during open heart surgery [11]. On the other hand, a major shift of intestinal microbiota to pathogenic species coinciding with reduced microbial diversity occurs in both systemic inflammatory response syndrome and neonatal sepsis patients [12]. Both Gram-negative bacteria (e.g., *Escherichia coli*, *Klebsiella*, *Enterobacter* spp.) and Gram-positive bacteria (e.g., *Staphylococcus*, *Enterococcus*, *Streptococcus* spp.) play a role in bacteremia or sepsis in neonates [13], infants [14], and adults [15]. Thus, a compromised gut barrier can lead to bacterial translocation and bacteremia, which could lead to systemic inflammation and, in susceptible patients, to sepsis, septic shock, and circulatory collapse, with or without multiple organ dysfunction syndrome (MODS). The role of local events in the intestines, the importance of the gut–liver axis, the contribution of biliary and pancreatic enzymes, and, finally, the gut–lung connection are discussed in the sections below.

## Gastrointestinal failure

**Fig. 1:**
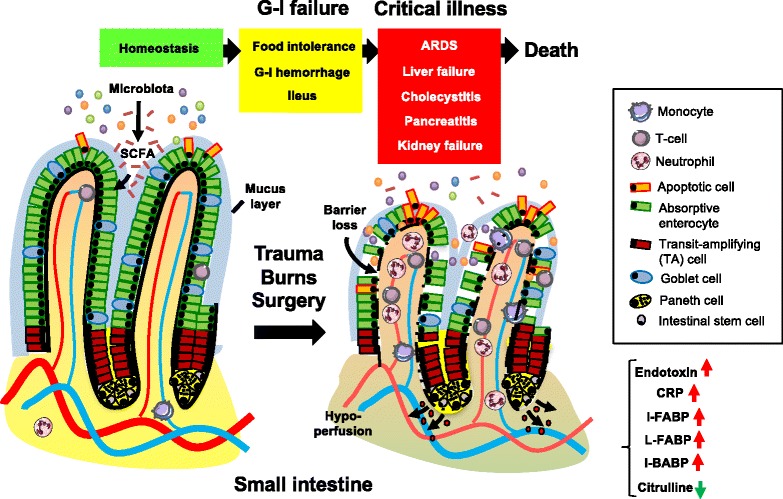
Pathogenic events in the course of gastro-intestinal failure. Potential toxic components in the small intestine are contained in the lumen under homeostatic conditions, whilst enterocytes allow the uptake of nutrients such as short-chain fatty acids (*SCFA*) across the epithelial barrier. In the critically ill patient suffering from major trauma or burns or undergoing surgery, protective mechanisms that maintain the gut barrier fail due to circulatory and neuroendocrine dysregulation. This results in gastro-intestinal (*G-I*) failure, which coincides with clinical signs such as oral intolerance, gastrointestinal hemorrhage, or ileus. G-I failure is a progressive clinical syndrome in which an early stage of predominantly gastrointestinal symptoms may be followed by extraintestinal derangements, such as acute respiratory distress syndrome (*ARDS*), liver failure, cholecystitis, pancreatitis, or kidney failure. G-I failure is associated with epithelial denudation, villus shortening, and inflammatory cell infiltration in the small intestine at the tissue level. Finally, various clinical biomarkers may aid in the diagnosis of G-I failure, including plasma or urinary levels of intestinal-type fatty acid-binding protein (*I-FABP*), liver-type fatty acid-binding protein (*L-FABP*), ileal bile acid-binding protein (*I-BABP*) or citrulline. *CRP* C-reactive protein

The aforementioned deleterious events do not always remain indolent and may lead to the poorly defined clinical entity of G-I failure. Its symptoms include food intolerance, G-I hemorrhage, and ileus. In more severe cases, G-I failure may lead to liver failure, cholecystitis, and pancreatitis [16, 17]. Postoperative patients frequently experience intestinal failure of various degrees of severity [18]. A grading system of acute G-I injury was recently proposed with increasing severity from grade I (risk of developing G-I dysfunction or failure), grade II (G-I dysfunction), grade III (G-I failure), to grade IV (G-I failure with severe impact on distant organ function) [19]. Early diagnosis of G-I failure is challenging as problems with enteral feeding, including vomiting, delayed gastric emptying, and diarrhea, can occur in up to 50 % of critically ill patients [20]. Since enteral nutrition has beneficial effects on the gut barrier, parenteral feeding may lead to further deterioration of G-I physiology. Gut dysfunction and G-I failure are associated with prolonged intensive care unit (ICU) stay and increased mortality [21, 22]. In fact, the presence of three or more G-I symptoms (high gastric residual volume, absent bowel sounds, vomiting, diarrhea, bowel distension, and G-I bleeding) on the first day of ICU admission is associated with a threefold increase in mortality [23]. To aid in the diagnosis of G-I failure, plasma or urinary levels of intestinal fatty acid-binding protein (I-FABP), liver fatty acid-binding protein (L-FABP) and ileal bile acid-binding protein (I-BABP), and/or citrulline can be helpful [24, 25]. I-FABP, L-FABP, and I-BABP are reliable biomarkers of enterocyte damage and/or loss and their urinary or plasma levels increase during intestinal injury. Plasma levels of citrulline represent enterocyte mass and/or functionality [26]. In the course of G-I failure, plasma citrulline levels would therefore be decreased and are indicative of a loss of the gut barrier [24, 27]. Indeed, low citrulline levels are associated with elevated serum C-reactive protein (CRP) levels, an increased rate of nosocomial infections, and higher mortality in critically ill patients [28]. A recent report showed that I-FABP can serve as a biomarker for the intensity of intestinal handling during surgery [29]. These serum markers also demonstrated that gut barrier loss is prevalent in patients that underwent non-abdominal surgery [30]. Finally, the systemic release of I-FABP in the course of surgery coincided with endotoxemia [31], whereas the administration of endotoxin in healthy subjects induced an increased intestinal permeability [32]. Together, these data further support the association between gut barrier dysfunction and systemic inflammation, as shown in Fig. 1.

## Early event: loss of the gut barrier

The gut barrier must be permissible to allow uptake of essential nutrients, but also needs to retain harmful substances that are only micrometers away from the circulation. These include microbial substrates (e.g., LPS), dietary components (e.g., free fatty acids (FFAs)) and digestive enzymes produced by the exocrine pancreas. The main etiological factors that contribute to gut barrier loss after surgery are splanchnic hypoperfusion (including ischemia/reperfusion damage) [30], decreased gut motility, and hypoxia. These processes are under control of neuronal and endocrine effector arms, in addition to local control mechanisms, which also affect the microcirculation. Hypotension associated with systemic inflammatory response syndrome usually results in shunting of blood from the splanchnic vessels to the central circulation. The perfusion of the small intestine operates within tight margins; the lack of autoregulatory mechanisms results in a hypersensitivity to variations in blood pressure. The combination of hypoperfusion and hypoxia can further exacerbate the deleterious effects on intestinal tissue integrity [33]. Hypoperfusion and ischemia/reperfusion (I/R) injury are partners in crime with regard to intestinal pathology [34], even though the latter displays distinct features. Importantly, short periods of I/R did not induce intestinal inflammatory responses in human [35], suggesting that a certain threshold of injury is required for the instigation of G-I failure. An adequate microcirculation is also required for optimal blood flow to tissues, particularly in the G-I tract. Various clinical tools are available to monitor the microcirculation, including orthogonal polarization spectral and sidestream darkfield imaging [36]. Finally, the severity and/or duration of intestinal ischemia are directly correlated with the degree of gut barrier loss and endothelial dysfunction [37]. The reticulo-endothelial system in the liver provides a fail-safe mechanism to filter out any toxic translocation products from the portal circulation that leaked from the intestines. However, Kupffer cells (KCs) of the liver—which are key players in the reticulo-endothelial system—appear to play a dual role in the regulation of inflammation. Whereas KCs are an important source of pro-inflammatory cytokines in systemic inflammation [38], they may also play an anti-inflammatory role under these conditions [39, 40]. Finally, a portion of potentially deleterious elements can also find a shortcut to the circulation via the mesenteric lymph.

## The gut–liver axis in systemic inflammation

The portal vein works as a major expressway from the spleen and G-I tract to the liver, accounting for approximately 70 % of the hepatic blood supply. This anatomical situation makes the liver a central location for clearing systemic bacterial infections and for maintaining immune system homeostasis. Lining the hepatic sinusoids, KCs are the first macrophage population to encounter bacteria or microbial products derived from the intestine. However, the function of KCs is compromised in patients with advanced liver disease and, despite the threatening consequences of impaired liver function, systemic infections are the main cause of death in these patients [41]. Many of these infections are initiated by translocation of intestinal bacteria and usually result in bacteremia and, in more severe cases, sepsis [42]. Bacterial translocation can be demonstrated by analysis of mesenteric lymphatics or portal vein blood samples. It is important to point out here that, in a classic study, portal vein sampling in trauma patients undergoing laparotomy did not provide evidence for bacterial translocation by blood cultures [43]. Subsequent studies with trauma patients confirmed that blood cultures generally failed to show bacterial growth [44, 45]. However, more sensitive methods, such as immunostaining for *E. coli* beta-galactosidase [44] or electron microscopy [45], provided direct evidence for bacterial translocation to mesenteric lymph nodes (MLNs) in most patients. While the presence of bacteria in MLNs as a pathological event has been debated [46, 47], multiple studies have shown that positive cultures from MLN samples obtained from laparotomy patients occurred in 10–15 % of patients, which correlated with an increased risk of postoperative sepsis [48, 49] or postoperative infection [50].

Both human and animal model studies have provided more mechanistic details on gut–liver crosstalk (Table 1). Bacteremia increases the risk of spontaneous bacterial peritonitis, which occurs in one out of five hospitalized cirrhotic patients [51]. At the same time, microbiota-driven inflammation can also aggravate liver disease. For example, alterations in colonic microbiota are associated with endotoxemia and inflammation in patients with hepatic encephalopathy, a complication of liver cirrhosis [52, 53]. Gut bacteria dysbiosis may also contribute to postoperative infections and organ rejection after liver transplant [54]. In fact, acute liver rejection in rats is accompanied by alterations in gut microbiota, impaired integrity of the intestinal barrier, bacterial translocation, elevation of plasma endotoxin levels, and a systemic inflammatory response [55, 56]. Gut microbiota dysbiosis can also trigger local inflammation in the liver and promote the progression from moderate liver disease to steatohepatitis [57]. A recent study showed that microbiota-dependent activation of the chemokine receptor CX3CR1 in intestinal macrophages is crucial for maintaining intestinal homeostasis and barrier integrity and, therefore, for controlling steatohepatitis progression [58]. Since advanced liver disease usually leads to circulatory abnormalities (portal hypertension, splanchnic vasodilation), these events may constitute a vicious circle. Damage to the intestinal barrier leads to bacterial translocation and thus liver inflammation and liver dysfunction, leading to exacerbation of circulatory abnormalities and causing further intestinal injury [59].

**Table 1:** Gut-liver crosstalk in systemic inflammation

Major finding	Study type	Reference
Bacteremia due to bacterial translocation is associated with an increased risk of bacterial peritonitis	Human	[51]
Hepatic encephalopathy is associated with changes in the intestinal microbiota	Human	[52]
Acute liver rejection is associated with changes in intestinal microbiota, loss of gut barrier, and enhanced systemic inflammation	Rodent	[55, 56]
CX3CR1 signaling by intestinal macrophages regulates steatohepatitis	Rodent	[58]
A hepatic vascular and phagocytic network functions as a “secondary firewall” to filter escaped gut commensals	Rodent	[60]
Liver cirrhosis is associated with increased inflammasome activation in ascitic fluid macrophages	Human	[61]
Translocation of bacterial DNA in liver cirrhosis is associated with enhanced systemic inflammatory activity	Human	[62, 63]
Hepatic STAT3 signaling protects against systemic inflammation caused by polymicrobial sepsis	Rodent	[72]
Hepatic gp130-STAT3 signaling induces myeloid-derived suppressor cells that protect against polymicrobial sepsis	Rodent	[73]
Hepatic STAT3 signaling regulates cellular and humoral pulmonary immunity against bacterial infection	Rodent	[74]
The liver enhances and protects against systemic inflammation through Kupffer cell-mediated cytokine production and detoxification by parenchymal cells, respectively	Rodent	[76]
Kupffer cells aggravate lung damage in acute pancreatitis	Rodent	[78, 79]

Summary of key studies that addressed the bidirectional gut–liver crosstalk in the pathogenesis of systemic inflammation. The type of study (animal model or human) is indicated

*STAT3* signal transducer and activator of transcription 3

Emphasizing the relationship between microbiota and the immune response in the liver, recent data suggest that the liver remains sterile when the intestine is healthy. However, the liver becomes an important “secondary firewall” when commensal bacteria penetrate the mesenteric circulation [60]. Importantly, not only viable bacteria but also abnormal amounts of microbial products (e.g., LPS or bacterial DNA) can breach the intestine and arrive at the liver or the ascitic fluid. These events have been associated with activation of the inflammasome complex [61], systemic inflammatory responses [62, 63], and acute-on-chronic liver failure [64]. The microbial antigens are recognized by pattern recognition receptors (PRRs), namely Toll-like receptors (TLRs), which in the liver are expressed on KCs, hepatic stellate cells, intrahepatic lymphocytes, dendritic cells, endothelial cells, and hepatocytes [65]. TLR-mediated activation of hepatic stellate cells is associated with the production of proinflammatory cytokines [66] and the development of liver fibrosis [67]. KCs normally produce proinflammatory cytokines and promote immune cell recruitment after TLR stimulation; however, continuous stimulation of KCs with low levels of LPS induces LPS tolerance and release of anti-inflammatory cytokines such as interleukin (IL)-10 [68]. Furthermore, in the healthy liver LPS is detoxified by both KCs and hepatocytes and rapidly loses its biological activity. Thus, it has been speculated that activation of TLR4 on KCs might be a common event and play a role in immune homeostasis, whereas activation of other PRRs, like NOD-like receptors, may be more frequent in infection [54]. Several studies also pointed to crosstalk between KCs and migrating neutrophils. Bactericidal neutrophils migrate to the liver sinusoids during endotoxemia and sepsis and, along with KCs, form the leading force in the elimination of bacteria in the liver [69]. In turn, elimination of neutrophils is necessary to resolve inflammation and previous studies suggested that KCs ingest and eliminate neutrophils in the liver sinusoids after microbial clearance [70]. These interactions may play a critical role in downregulating pro-inflammatory cytokine and chemokine production by KCs [71].

The liver also maintains immune homeostasis in other organs, with an important role for hepatocyte signal transducer and activator of transcription 3 (STAT3) during sepsis and pneumonia. Hepatic STAT3 activity was necessary to prevent an excessive systemic inflammatory response and attenuate lethality after cecal ligation and puncture-induced sepsis [72]. STAT3-mediated protection in this model of polymicrobial sepsis was attributed to the serum amyloid A and CXCL1-dependent mobilization of myeloid-derived suppressor cells [73]. Similarly, a recent study showed that pre-existing liver STAT3 activation modulates host immune responses in a two-hit model of endotoxemia followed by bacterial lung challenge [74]. Mice with hepatocyte-specific STAT3 deletion showed reduced concentrations of acute phase response proteins serum amyloid A and serum amyloid P, impaired alveolar macrophage reactive oxygen species generation, higher lung and blood bacterial loads, and increased mortality in this model [74]. Thus, physiological amounts of bacterial ligands arriving at the liver from the gut can contribute to maintain systemic immune homeostasis through the induction of STAT3 activity. However, gut alterations and excessive microbiota-dependent liver inflammation may shift this balance towards a more proinflammatory phenotype, leading to damage in remote organs such as brain, lung, pancreas, and heart [75]. A good example of this crosstalk is the gut–liver–lung axis during acute pancreatitis, leading to the acute respiratory distress syndrome (ARDS) and MODS. In this scenario, severe pancreatitis triggers intestinal barrier dysfunction and gut inflammation. Translocated microbial products and inflammatory mediators produced in the gut (e.g., tumor necrosis factor (TNF)-α, IL-6, and IL-1β) then arrive at the liver via the portal vein and activate KCs, which produce more pro-inflammatory cytokines that amplify the inflammatory response [76]. These cytokines released by the liver are then transported via the systemic circulation to the lung, where they cause acute hemorrhagic necrosis of lung epithelial cells and activation of pulmonary monocytes and macrophages, ultimately contributing to ARDS and MODS [77, 78]. Indeed, inhibition of KCs has been shown to reduce pancreatitis-associated remote organ injury [78, 79]. Together, these studies suggest that failure of the gut barrier may constitute a fatal event in patients with end-stage liver disease and that the “gut–liver inflammation” axis may play an important role in the balance between tolerance and systemic inflammation in critical illness.

## Intestinal damage induced by bile acids

In addition to the liver serving as a secondary firewall, there is strict compartmentalization of secretions of the biliary tract and exocrine pancreas. Bile acids and pancreatic enzymes are excreted through the pancreatic duct to promote the digestion and uptake of lipids, carbohydrates, and amino acids. Primary bile acids are synthesized in the liver as derivatives of cholesterol, e.g., cholic acid and chenodeoxycholic acid. These are converted by the intestinal microflora to yield secondary bile acids, deoxycholic acid (DCA), lithocholic acid, and ursodeoxycholic acid (UDCA) [80]. Bile is released in the duodenal lumen in response to cholecystokinin (CCK) produced by duodenal cells postprandially [81]. The bile acids are reabsorbed with dietary lipids and returned to the liver via the enterohepatic circulation, whereas saturated digestive enzymes are normally excreted and eliminated [80]. However, under certain conditions bile acids may pave the way for proteases to breach the gut barrier and cause systemic inflammatory responses. Importantly, the different types of bile acids have differential effects on the gut barrier. For example, whereas cholic acid, DCA, and chenodeoxycholic acid showed disruptive effects on the intestinal epithelial barrier in vitro, this was not the case for UDCA [82]. Furthermore, exposure of epithelial cells to concentrations of DCA corresponding to high-fat diets disrupted the epithelial barrier in vitro and in vivo. In contrast, concentrations of DCA typically found in low-fat diets did not affect the epithelial integrity [83]. In this context, the effects of enteral nutrition versus total parenteral nutrition on the gut barrier in critically ill patients are highly relevant. Total parenteral nutrition increases the gut permeability, which in addition to the effects of bile acids may involve a variety of mechanisms [84]. Others showed protective effects of lipid-rich enteral feeding on the gut barrier in experimental shock, which was reversed by CCK receptor antagonists [85]. These data suggest an intrinsic protective effect of enteral nutrition on the gut barrier. Thus, even though enteral feeding stimulates the release of bile acids in the gut lumen that could potentially damage the epithelial lining, its advantageous effects on the gut barrier appear to be dominant.

## Intestinal and extra-intestinal injury caused by pancreatic enzymes

**Fig. 2:**
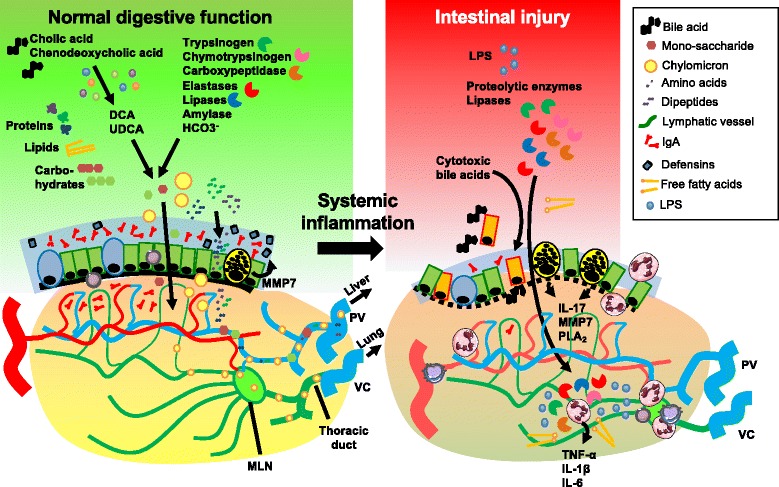
Cellular and molecular players in intestinal injury caused by systemic inflammation. A large variety of pancreatic and hepatobiliary products are involved in the complex digestive function of the small intestine, e.g., proteolytic enzymes, lipases, amylases, bicarbonate, and primary bile acids. The primary bile acids (cholic acid, chenodeoxycholic acid) are converted to secondary bile acids (e.g., lithocholic acid, DCA, UDCA) by the microbiota. The digestive enzymes derived from the host or microbiota ensure the breakdown of macromolecules to soluble nutrients, which is followed by uptake and transport to the portal circulation or intestinal lymph tract. While the intestinal microflora are crucial for these digestive functions in the lumen, their quantity and topography are tightly controlled by the thick mucus layer that contains secretory IgA and antimicrobial peptides (e.g., defensins) produced by mucosal immune cells and the epithelium. In critical illness, circulatory derangement results in gut barrier loss and the translocation of digestive enzymes, cytotoxic bile acids, free fatty acids (FFAs), and microbial substrates to the submucosa, which exacerbates local and systemic inflammatory reactions. The intestinal epithelium is also an important source of pro-inflammatory mediators that are released into the circulation, including IL-17, lipid mediators produced by phospholipase A2 (*PLA*_*2*_), and antimicrobial peptides derived from Paneth cells. These pathogenic events also pave the way for cytotoxic components to directly leak from the intestinal lumen to mesenteric lymph vessels, which constitutes the gut–lymph–lung axis. *MLN* mesenteric lymph node, *MMP* matrix metalloproteinase, *PV* portal vein, *VC* vena cava

The local and systemic effects of pancreatic enzymes in the critically ill patient have recently regained attention. Acinar cells of the exocrine pancreas secrete a variety of enzymes that, upon activation in the duodenal lumen, can degrade proteins (trypsinogen, chymotrypsinogen, carboxypeptidase, elastases), lipids (pancreatic lipase, phospholipase), and sugars (pancreatic amylase). Bicarbonate (HCO_3_^−^) is produced by ductal cells. This pancreatic juice is secreted in response to CCK. However, during intestinal ischemia these proteases can contribute to degradation of the protective mucus layer and epithelial tight-junctions, leading to an elevated gut permeability and penetration of serine proteases of the gut wall [86]. This was first demonstrated by pancreatic duct ligation, which reduced gut barrier failure in an experimental model of hemorrhage-associated shock [87]. Prevention of pancreatic enzyme influx in the intestinal lumen resulted in reduced degradation of the mucus layer, less toxicity to endothelial cells, and reduced activation of circulating neutrophils [87]. The toxic effects of pancreatic enzymes in vivo, including trypsin, chymotrypsin, elastase, amylase, and lipase, are potentiated when the mucus layer is already compromised [88]. Conversely, the addition of a mucus layer to IEC monolayers reduced the disruptive effects of trypsin on the gut barrier [89]. Importantly, the translocation of active proteases to the circulation is associated with an increased risk of multi-organ failure [90], most likely caused by local autodigestive processes in the gut that liberate tissue or microbial factors that turn on systemic inflammatory responses [91]. These data suggest that pharmacological inhibition of pancreatic enzymes could abrogate these pathophysiological events and ameliorate circulatory derangement in critical illness. A recent publication demonstrated this concept in three models of experimental shock in rats—hemorrhagic shock, peritonitis, and endotoxin shock—with a focus on serine proteases. All models of shock resulted in increased protease activity in the gut wall. Conversely, intraluminal administration of protease inhibitors (ANGD, tranexamic acid, and aprotinin) dramatically reduced tissue damage in the small intestines as well as distant organs (heart, lung), which was associated with significantly improved survival [92]. Notably, direct and invasive administration of protease inhibitors was required to yield high intraluminal concentrations in the gut [92], which limits its direct translation into clinical practice. Alternatively, continuous delivery of protease inhibitors via enteral feeding has been shown to be successful in one case of septic shock [93]. Together, these data suggest that under ischemic conditions in the gut, pancreatic enzymes can mediate deleterious local and systemic effects in the critically ill patient, as summarized in Fig. 2.

## The gut–lung connection: mesenteric lymph

A direct anatomical link between the gut, systemic circulation, and distant organs is provided by the lymphatic vessels from the intestines. After feeding, the mesenteric lymph is enriched for lipids (chylomicrons), fat-soluble vitamins, and a variety of other lipophilic macromolecules. These afferent lymphatic vessels drain from the intestinal villus tips to MLNs and ultimately into the thoracic duct, heart, and the pulmonary circulation. Within this network, MLNs are the site where luminal antigens are filtered and taken up by antigen-presenting cells (macrophages, dendritic cells), which can direct adaptive immune responses. The cellular players of this specialized mucosal immune system shuttle between MLNs and the lamina propria and are programmed for successful compartmentalization of the commensal microflora. One of the mechanisms involved in this process is the deposition of secretory IgA in the mucus layer [94], an adaption to the unusually large bacterial load of the G-I tract.

Mesenteric lymph avoids the portal circulation and thus bypasses the reticulo-endothelial system in the liver, i.e., the secondary firewall mediated by KCs. Any unfiltered luminal constituents, such as endotoxins and pancreatic enzymes, in addition to locally produced cytokines and activated immune cells that exit the MLN, are able to directly leak to the circulation. Cytotoxic factors from mesenteric lymph will make their first pass through the pulmonary circulation. Direct toxic effects on the pulmonary endothelium can then cause acute lung injury, culminating in ARDS [95]. This is known as the gut–lymph–lung axis. Indeed, abrogation of mesenteric lymph drainage prevented hemorrhagic shock-induced endothelial hyperpermeability and lung damage [96]. Furthermore, real-time cross-transfusion of mesenteric lymph from donor rats with trauma/hemorrhagic shock to naïve recipients resulted in marked lung injury and local neutrophil accumulation in these recipients [97]. Virtually all studies that have addressed the components of mesenteric lymph used animal models (Table 2); there is little information regarding the toxic components of post-shock mesenteric lymph in human [98]. Bacteria or microbial products do not play a role in this phenomenon [99]. Rather, various acute-phase proteins, possibly produced by the intestinal epithelium [100], in addition to pro-inflammatory lipid mediators [101], and in particular lipase-generated FFAs [102], are likely causative agents. Their cytotoxicity appears to be determined by the FFA-to-protein ratio in mesenteric lymph, as the addition of albumin—a lipid scavenger—reversed their effects [102]. Serum albumin precursor was the most (approximately eightfold) upregulated protein in post-shock mesenteric lymph [100], which could therefore be part of a compensatory mechanism to inhibit lipid cytotoxicity [103]. On the other hand, glycosylated albumin in post-shock mesenteric lymph has been associated with intrinsic cytotoxicity [104]. Thus, albumin can have both protective and deleterious effects on lung injury.

**Table 2:** Gut–lung crosstalk

Major finding	Study type	Reference
Cross-transfusion of mesenteric lymph from donors after shock/trauma induces acute lung injury in recipients	Rodent	[97]
Post-shock mesenteric lymph contains increased amounts of bioactive lysophospholipids and PUFAs	Rodent	[101]
Post-shock mesenteric lymph contains increased amounts of free fatty acids	Rodent	[102]
The detrimental effects of post-shock mesenteric lymph can be reversed by the addition of albumin	Rodent	[103]
Inhibition of PLA_2_ inhibits the cytotoxic activity of post-shock mesenteric lymph	Rodent	[105]
Levels of Paneth cell-derived α-defensin 4 (an antimicrobial peptide) are increased in post-shock mesenteric lymph	Rodent	[107]
Endogenous alarmins are abundantly released from the intestines in post-shock mesenteric lymph	Rodent	[113]

Pertinent studies that addressed the interactions between intestinal pathology and lung injury

*PLA*_*2*_ phospholipase A2, *PUFA* poly-unsaturated fatty acids

Another candidate to mediate the toxic effects of mesenteric lymph is phospholipase A2 (PLA_2_), an enzyme that generates lipid mediators (eicosanoids, prostaglandins) [105]. Importantly, high levels of PLA_2_ are synthesized and secreted by Paneth cells in the intestine [106]. Another antimicrobial protein produced by Paneth cells that was found to be increased in toxic mesenteric lymph was α-defensin 4 [107]. Together, this suggests a potential detrimental role for Paneth cell products in the course of systemic inflammation and/or shock, which are distributed via the intestinal lymphatic system to the circulation. Indeed, mice deficient for matrix metalloproteinase 7 (MMP7), the enzyme involved in posttranslational activation of Paneth cells products, were protected against systemic LPS-induced lethality [108]. Activation of Paneth cells and transport of Paneth cell products, including pro-inflammatory IL-17A, by intestinal macrophages to the liver have been previously demonstrated in systemic inflammation [109]. IL-17 is an important activator of neutrophils and Paneth cells were the main producers of IL-17 in an experimental model of TNF-α-induced intestinal injury and shock [110]. Furthermore, liver I/R injury and its associated systemic inflammation resulted in significantly increased IL-17 levels in the portal venous blood, which was associated with massive Paneth cell degranulation in the gut and hepatic, intestinal, and renal injury. Pharmacological or genetic approaches to abrogate Paneth cell function reversed these effects [111]. Similarly, genetic knockout for IL-17A also prevented intestinal damage in this I/R model [112]. These findings further demonstrate a central role for the gut as a driving force in systemic inflammation (Fig. 2).

Finally, increased levels of alarmins that are generally released after tissue injury were also found to be elevated in post-shock mesenteric lymph [113]. These substrates are endogenous TLR4 ligands and mediate immunostimulatory effects via the activation of nuclear factor kappa B (NF-kB). Mice with genetic mutations in the receptors or adapter molecules from the TLR4 signaling pathway were protected against post-shock mesenteric lymph-mediated lung injury [114]. These findings are consistent with the “danger model” that states that certain endogenous ligands signal the presence of tissue injury to the host via PRRs [115], including TLR.

## Conclusions

**Fig. 3:**
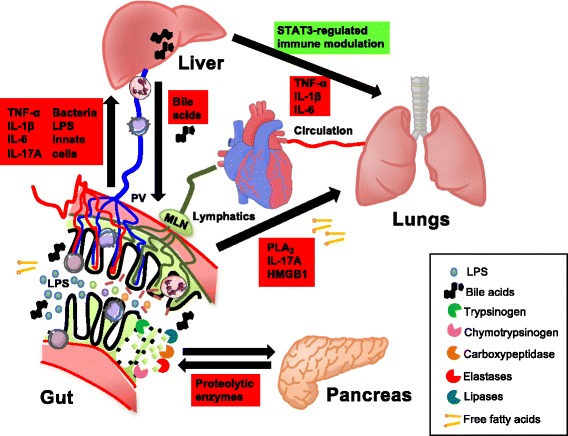
Direct and indirect interactions between the gut and other organs in systemic inflammation. Luminal components of the small intestine can spread to the circulation via the portal vein (*PV*) and liver. This includes pro-inflammatory constituents such as lipopolysaccharide (*LPS*), bacterial DNA, whole bacteria, other bacterial products, and free fatty acids (FFAs). Toxic components of the gut lumen, including FFAs, inflammatory products of phospholipase A2 (*PLA*_*2*_), pro-inflammatory cytokines (e.g., *IL-17*), and damage-associated molecular substrates such as high mobility group box 1 protein (*HMGB1*), can reach the pulmonary circulation via mesenteric lymph. Bile acids from the liver, including cholic acid, DCA, and chenodeoxycholic acid, mediate cytotoxic effects on intestinal epithelial cells. STAT3 signaling in Kupffer cells (KCs) of the liver maintains tolerance under homeostatic conditions, whereas KCs produce high levels of pro-inflammatory cytokines in systemic inflammation with toxic effects on the lung parenchyma. Finally, activated serine proteases, elastases, and lipases produced by the pancreas can cause local tissue destruction and activation of immune cells in intestinal tissues, leading to exacerbated systemic inflammatory responses

In conclusion, the mesenteric lymph is another route for immunostimulatory proteins to reach the systemic circulation after intestinal injury and post-shock mesenteric lymph is particularly toxic to the pulmonary microvasculature. Gut-derived toxic factors that leak from MLNs, including products of pancreatic enzymes, endogenous danger signals, and Paneth cell products, most likely partner up to exert these detrimental effects. All the aforementioned interactions are summarized in Fig. 3. Finally, basic insights into the intimate relationship between the G-I tract and the systemic inflammatory system are expected to lead to more efficacious treatment modalities for critical illness in the future.

## Abbreviations

ARDS: Acute respiratory distress syndrome
CCK: Cholecystokinin
DCA: Deoxycholic acid
FFA: Free fatty acid
G-I: Gastro-intestinal
GPR: G-protein-coupled receptor
I-BABP: Ileal bile acid-binding protein
ICU: Intensive care unit
IEC: Intestinal epithelial cell
I-FABP: Intestinal fatty acid-binding protein
IL: Interleukin
I/R: Ischemia/reperfusion
KC: Kupffer cell
L-FABP: Liver fatty acid-binding protein
LPS: Lipopolysaccharide
MLN: Mesenteric lymph node
MODS: Multiple organ dysfunction syndrome
PLA_2_: Phospholipase A2
PRR: Pattern recognition receptor
SCFA: Short-chain fatty acid
STAT3: Signal transducer and activator of transcription 3
TLR: Toll-like receptor
TNF: tumor necrosis factor
UDCA: Ursodeoxycholic acid
